# The Effect of Enalapril, Losartan, or Not Antihypertensive on the Oxidative Status in Renal Transplant Recipients

**DOI:** 10.1155/2022/5622626

**Published:** 2022-03-10

**Authors:** Jorge Andrade-Sierra, Mónica Lizbeth Morales-Guillén, Andrés García-Sánchez, Elodia Nataly Díaz-de la Cruz, José Ignacio Cerrillos-Gutiérrez, Enrique Rojas-Campos, Alejandra Guillermina Miranda-Díaz

**Affiliations:** ^1^Nephrology and Transplantation Division, UMAE, National Medical Center of the West, Mexican Institute of Social Security, Guadalajara, Jalisco, Mexico; ^2^Department of Physiology, University Center for Health Sciences, University of Guadalajara, Mexico

## Abstract

The clinical and biochemical improvement observed in kidney transplant (RT) recipients is remarkable. The correct functioning of the allograft depends on various factors such as the donor's age, the alloimmune response, the ischemia-reperfusion injury, arterial hypertension, and the interstitial fibrosis of the allograft, among others. Antihypertensive drugs are necessary for arterial hypertension patients to avoid or reduce the probability of affecting graft function in RT recipients. Oxidative stress (OS) is another complex pathophysiological process with the ability to alter posttransplant kidney function. The study's objective was to determine the effect of the administration of Enalapril, Losartan, or not antihypertensive medication on the oxidative state in RT recipients at the beginning of the study and one year of follow-up. All patients included in the study found significant overexpression of the oxidative damage marker to DNA and the antioxidant enzymes superoxide dismutase (SOD) and glutathione peroxidase (GPx). In contrast, it was found that the determination of the total antioxidant capacity decreased significantly in the final determination at one year of follow-up in all the patients who ingested Enalapril and Losartan. We found dysregulation of the oxidative state characterized mainly by oxidative damage to DNA and a significant increase in antioxidant enzymes, which could suggest a compensatory effect against the imbalance of the oxidative state.

## 1. Introduction

Chronic allograft dysfunction (CAD) is a progressive and irreversible entity and is a cause of long-term kidney transplant failure [1–3]. The mechanisms of damage that contribute to CAD involve immunological [4] and nonimmunological [3] aspects, which conditions the formation of interstitial fibrosis and tubular atrophy (IFTA) [5]. On the other hand, the oxidative stress (OS) characterized by the imbalance between the generation of oxidative compounds and the antioxidant defense mechanisms could be resulted in allograft damage, especially when immunosuppression in renal transplantation is based on the use of calcineurin inhibitors (CNI) inducing endothelial dysfunction, hypertension, and increased renal nephrotoxicity, characterized by renal vasoconstriction, interstitial fibrosis, and arterial hypertrophy [6–9]. The generation of oxidative compounds is physiologically essential as part of the defense mechanism against the invasion of microorganisms, malignant cells, tissue repair, and healing. When the deregulated activation of OS occurs, it can cause vascular damage favoring the progression of atherosclerosis due to oxidative damage directly to cellular components, leading to impaired cell function, aging, and activation of apoptosis [10]. Lipoperoxidation products (LPO) damage lipoproteins present in the cell membrane, leading to the formation of toxic reactive aldehydes and promoting more significant lipid peroxidation, ultimately affecting many lipid molecules [10]. On the other hand, the 8-hydroxy-2′-deoxyguanosine (8-OHdG) is a sensitive and specific marker of oxidative damage to DNA [11].

Various therapeutic interventions are used to minimize kidney graft damage associated with CNI including the use of angiotensin-converting enzyme (ACE) inhibitors and angiotensin receptor blockers (ARBs) [12–14]. ACE inhibitors or ARBs are drugs with potent antihypertensive and renoprotective effects that are underutilized because it can cause elevated potassium and deleterious effect on renal hemodynamics with elevated serum creatinine (sCr) levels at some RT recipients, in addition to contradictory results in patients and graft survival in RT [15–17]. Nevertheless, the angiotensin II induces OS *in vitro* and *in vivo* and selectively blocks AT1 recipients ameliorated nonhemodynamic effects (reduction of OS) in renal nephrotoxicity due to CNI [18, 19]. Likewise the used of ACE inhibitors may potentiate protective mechanisms against long-term complications of CNI treatments (reducing effects on OS, fibrogenesis, and chronic rejection) [6].

The study's objective was to determine the effect of the administration of Enalapril, Losartan, or not antihypertensive on the oxidative state in RT recipients at the beginning of the study and one year of follow-up.

## 2. Patients and Methods

An open, randomized clinical trial was conducted with a control group. It was carried out in the Transplant Division of the High Specialty Medical Unit of the Hospital de Especialidades, Centro Médico Nacional de Occidente of the Mexican Institute of Social Security in Guadalajara, Jalisco, Mexico. The sample size was based on the formula to evaluate mean differences for clinical trials [20]. Three study groups were formed; thirteen patients with RT did not require any antihypertensive. Thirteen RT patients received Enalapril as an antihypertensive regimen. Thirteen RT patients received Losartan as an antihypertensive regimen in the posttransplant period. Recipients of a RT, from a living related donor (DVR) or living unrelated donor (DVNoR), agreed to participate and signed the letter of consent under information.

Patients who received an RT from a deceased donor, from a donor >55 years, with renal comorbidities at the time of the study (urolithiasis, infections, and diabetes), with blood dyscrasias, second transplantation, treatment with nonsteroidal anti-inflammatory drugs, statins, spironolactone, and pentoxifylline and patients with neurodegenerative processes or who withdrew the letter of consent under information were excluded.

Baseline determinations were made the day before RT and at one year of posttransplant follow-up. Type of donor and cold and hot ischemia time were recorded.

### 2.1. Immunosuppression Induction and Maintenance Scheme

TAC carried out the induction of immunosuppression at doses of 0.12 mg/kg/day divided into two doses, mycophenolate mofetil 2 g/day, and methylprednisolone 500 mg (day 0), polyclonal antibodies (thymoglobulin 1-1.5 mg/kg/day or humanized monoclonal antibodies interleukin 2R*α* basiliximab 20 mg at day 0 and 4 posttransplant). The immunization maintenance scheme was carried out by administering TAC at 0.1-0.2 mg/kg (the dose was modified according to serum levels). The target levels of TAC on days 1-30 posttransplantation were 9-15 ng/mL and from day 31 to 365 posttransplantation 8-10 ng/mL. The dose of mycophenolate mofetil was administered 1 g twice a day. Prednisone was administered at 1 mg/kg/day from the start of RT and was reduced to 0.1 mg/kg/day in the second month after transplantation [21].

### 2.2. Clinical and Biochemical Measurements

Patients were classified according to body mass index (BMI) 18.5-24.9 kg/m^2^ (normal weight), 25 to 29.9 kg/m^2^ (overweight), and ≥30 kg/m^2^ (obesity) [22], and the blood pressure was determined [23]. The biochemical data of plasma glucose and blood TAC levels were determined, maintaining levels of 9-15 ng/mL from 1-30 days and 8-10 ng/mL from 31-365 days after transplantation. The sCr levels were expressed in mg/dL and sCr clearance in 24?h urine was determined using the formula sCr debugging: sCr = sCr urinary (mg/dL) × 24 h urinary volume (mL) × sCr (mg/dL) × 1440 × body surface (m^2^).

The clearance of sCr was calculated with the formula: MDRD = GFR (mL/min × 1.73 m^2^) = 186 × CrS − 1.154 × (age) − ^0.203^ × (0.742 in case of being a woman)[24].

### 2.3. Oxidants

#### 2.3.1. Products of Lipoperoxidation (LPO)

The serum LPO was measured using an FR12 assay kit (Oxford Biomedical Research Inc®., Oxford, MI, USA). Plasma samples treated with N-methyl-2-phenylindole were centrifuged at 12,791 rpm for 10 min, and the supernatant was obtained. The supernatant was added to a microplate, and the absorbance was measured at 586 nm. The duplicate standard intra-assay CV was 6.4%.

#### 2.3.2. Nitric Oxide (NO)

Before the assay, plasma samples were deproteinized by adding zinc sulfate (6 mg of zinc sulfate was added to 400 *μ*L of the sample) and vortexed for one min, and the samples were centrifuged at 10,000 × *g* for 10 min at 4°C. For measuring NO, the kit NB98, Oxford Biochemical, Oxford, MI, USA, was used. The colorimetric signal was read at 540 nm. The duplicate standard intra-assay CV was 7.9%.

### 2.4. Antioxidants

#### 2.4.1. Superoxide Dismutase

The kit (SOD No. 706002, Cayman Chemical Company®, USA) was used. The serum samples were diluted at 1 : 2 in sample buffer before the colorimetric assay. Color development was read at a wavelength of 440 nm. The dilution factor was used to calculate the results. The duplicate standard intra-assay CV was 5.4%.

#### 2.4.2. Glutathione Peroxidase (GPx)

The assay kit GPx 703,102 was used (Cayman Chemical Company®, USA). Plasma samples (20 *μ*L) were added to a microplate of 96 wells with 70 *μ*L of buffer, 50 *μ*L of glutathione and glutathione reductase mixture, and 50 *μ*L of NADPH. The activity was obtained by measuring the absorbance decrease at 340 nm every min for 20 min. The activity is expressed as nmol/min/mL. The duplicate of positive control intra-assay CV was 1.5%.

#### 2.4.3. Total Antioxidant Capacity

The total antioxidant capacity (TAC) quantification was done following the Total Antioxidant Power Kit (No. TA02.090130, Oxford Biomedical Research®). The serum samples and standards were diluted at 1 : 40, and 200 *μ*L was placed in each well of a microplate. The concentration was expressed as mM equivalents of Trolox (an analog of vitamin E). The duplicate standard intra-assay CV was 5.7%.

### 2.5. Statistical Analysis

The results are presented in measures of central tendency and dispersion. The qualitative variables were determined in frequencies and percentages; the chi-square test was used when required. The results are expressed as mean ± standard deviation. The normality of data was determined, and the Shapiro-Wilk test was performed. For intragroup differences, the Wilcoxon rank test was used. The Kruskal-Wallis test was used for intergroup differences. The different letter denotes statistically significant difference using the Dunn-Bonferroni paired test. All *p* ≤ 0.05 values were considered statistically significant.

### 2.6. Ethical Considerations

The study adhered to the ethical principles for medical research in human beings stipulated in the Declaration of Helsinki 64th General Assembly, Fortaleza, Brazil, October 2013 and in the Belmont Report. The Standards of Good Clinical Practice and the International Conference on Harmonization were followed. The study was category III (risk more significant than the minimum). The following provision of the General Health Law in Mexico on health research, Title Two, of the Ethical Aspects of research in human beings, Chapter I, Article 17 Letter of Consent Under Information, was required with the signature of witnesses. The study was approved by the Local Ethics and Research Committee with registration 2018-1001-214, before Cofepris 17CI11 020 146 and before Bioethics with number 11 CEI 003 20188080. The clinical trial registration number is NCT05232370.

## 3. Results

The recipients of the three RT groups were young patients (*p* < 0.01). Kidney donors were <50 years. The male gender predominated in the three study groups. Age showed a difference in the group that did not require antihypertensive medication 29 ± 3.83 years vs. those treated with Enalapril 24.38 ± 1.61 years (*p* ≤ 0.01). The stature of the patients treated with Enalapril was greater than that of the other groups (*p* ≤ 0.01). The number of transfusions and the time of hot and cold ischemia were similar in the three study groups (Table 1).

### 3.1. Clinical Data

The patients who did not require antihypertensive medication and those who ingested Losartan gained weight in the one-year follow-up measurement. The BMI increased significantly in the final determination in those who ingested Losartan (*p* < 0.01). Patients with RT who did not require antihypertensive medication kept their blood pressure normal throughout the study. Thirteen patients who had elevated baseline systolic and diastolic blood pressure (134.62 ± 7.54 mmHg and 87.31 ± 4.34 mmHg) (*p* < 0.01) were administered with Enalapril. After one year of follow-up of the patients who ingested Enalapril, their blood pressure was significantly modified (systolic 127.8 ± 4 mmHg and diastolic 78.92 ± 1.5 mmHg, *p* < 0.01). The thirteen patients with baseline systolic blood pressure values of 140 ± 4.83 mmHg and diastolic 89.92 ± 5.48 mmHg (*p* < 0.01) were administered with Losartan. In the determination at one year of follow-up, the systolic blood pressure was modified to 131.31 ± 7 mmHg and diastolic to 80.77 ± 5.3 mmHg (*p* < 0.01). Glucose decreased its concentration in the group without antihypertensive medication in the final determination with 137.38 ± 80.42 mg/dL baseline vs. 90.46 ± 17.46 mg/dL final (*p* = 0.03). The sCr levels were similar in the three groups in the baseline determination. At the end of the study, an apparent decrease in sCr levels was shown in the three study groups. The patients who did not require antihypertensive medication obtained sCr levels in the baseline determination, 12.84 ± 3.77 mg/dL vs. 1.18 ± 0.15 mg/dL at one year of follow-up (*p* < 0.01). Those who ingested Enalapril had baseline sCr levels of 12.16 ± 4.63 mg/dL and final levels of 1.17 ± 0.21 mg/dL (*p* < 0.01). The baseline sCr levels in those who ingested Losartan were 13.38 ± 1.79 mg/dL and at one year of follow-up 2.6 ± 3.27 mg/dL (*p* < 0.01). Area concentration in the three groups was different (*p* = 0.02), the group without antihypertensive was 122.92 ± 37.74 mg/dL at baseline levels vs. 31.39 ± 6.68 mg/dL in the final determination (*p* < 0.01). In those who ingested Enalapril, the baseline urea determination was 155.59 ± 28.3 mg/dL vs. 30.82 ± 5.05 mg/dL at the end of follow-up (*p* < 0.01). For the group treated with Losartan, the baseline urea levels were 127.40 ± 32.11 mg/dL vs. 70.04 ± 72.94 mg/dL at the end of follow-up (*p* < 0.04). TAC levels were homogeneous in the three study groups between the baseline results and one year of follow-up (Table 2).

### 3.2. Oxidative Stress Markers

#### 3.2.1. LPO and Nitric Oxide Metabolites (NO)

Table 3 shows the results of the LPO levels with the significant increase in LPO between the basal levels 5.6 ± 2.61 mM, and the final levels 8.65 ± 1.09 mM (*p* ≤ 0.01) in patients who did not require antihypertensive medication can be seen. The LPO levels in the patients taking Enalapril and Losartan were similar between baseline and final results. NO metabolites did not show significant differences between the baseline and final results in the three study groups.

#### 3.2.2. Marker of Oxidative Damage to DNA

The baseline levels of the oxidative damage marker to DNA in patients who did not require antihypertensive drugs were 68.88 ± 11 ng/mL with a significant increase at one year of follow-up, 74.47 ± 0.39 ng/mL (*p* < 0.01). At one year of follow-up, the levels of those who ingested Enalapril increased significantly to 74.29 ± 0.63 ng/mL (*p* = 0.02) vs. baseline levels 52.94 ± 21.45 ng/mL. The same behavior was observed in the patients who ingested Losartan; in the baseline determination, 62.71 ± 19.6 ng/mL was obtained with a significant increase at one year of follow-up of 73.78 ± 1.88 ng/mL (*p* < 0.01). Significant intragroup differences were obtained (Table 3).

#### 3.2.3. Antioxidants

A significant increase in the enzyme superoxide dismutase (SOD) activity was observed in the three study groups. In patients who did not require antihypertensive medication, baseline levels were 0.3 ± 0.2 U/L and at the end of follow-up 1.17 ± 0.63 U/L (*p* < 0.01). The activity of the SOD enzyme in the basal Enalapril group was 0.39 ± 0.17 U/L vs. at one year of follow-up 1.31 ± 0.67 U/L (*p* < 0.01). The activity of the SOD enzyme at the end of the follow-up of those who ingested Losartan was 1.19 ± 0.46 vs. the basal activity of the enzyme 0.25 ± 0.21 U/L (*p* < 0.01) (Table 3).

The GPx enzyme only showed a significant increase in its activity for the group that did not require antihypertensive treatment with 343.76 ± 214.82 nmol/min/mL (baseline) vs. 518.15 ± 163 nmol/min/mL (final) (*p* = 0.03). GPx activity in those who ingested Enalapril and Losartan was similar.

The determination of the total antioxidant capacity (CAT) showed a significant decrease in its baseline concentration of 59.78 ± 1.89 *μ*M in patients who did not require antihypertensive treatment vs. the final determination 17.52 ± 3.4 *μ*M (*p* < 0.01). Patients who ingested Enalapril also decreased CAT levels between baseline 65.45 ± 18.99 *μ*M vs. the levels obtained at the end of the follow-up 14.2 ± 3.2 *μ*M (*p* < 0.01). A similar situation was observed in the patients who ingested Losartan where the baseline levels were 61.10 ± 24.07 *μ*M, and a significant decrease was observed at the end of the study 20.7 ± 5.3 *μ*M (*p* < 0.01) (Table 3).

## 4. Discussion

Significant overexpression of the oxidative DNA damage marker in the determination at one year of follow-up in the RT recipients who did not require antihypertensive medication is striking; the same phenomenon occurred in those who ingested Enalapril and Losartan. Changes in the epigenome and DNA methylation also could be implicated in each of the processes mentioned above to affect the kidney or other organ systems [25]. The increased expression of the oxidative damage marker to DNA can partially explain inflammation and cellular senescence, which could accelerate premature vascular aging at RT recipients [26–28]. Nuclear erythroid factor 2 (NRF2) signaling related to nuclear erythroid factor 2 (NRF2) and vitamin K play a crucial role in counteracting OS, DNA damage, senescence, and inflammation, thus activating NRF2 and supplementation with vitamin K which could offer an attractive therapeutic target in patients undergoing RT [29].

The antioxidant activity of the SOD enzyme was found to be significantly increased in the final determination in the three groups of patients, those treated with Enalapril and Losartan and those that did not require antihypertensive medication, possibly trying to compensate for the oxidative imbalance that occurs in the posttransplant period. Two antioxidants antagonize free radicals, including the antioxidant enzymes SOD, GPx, and catalase, and non-enzymatic antioxidants such as vitamin E, C, *β*-carotene, and coenzyme Q. The enzyme SOD is considered the first line of antioxidants capable of antagonizing the imbalance of the redox state. Following the present study, a significant increase in the activity of the SOD enzyme has recently been reported in patients undergoing hemodialysis [30]. The activity of the antioxidant enzyme GPx was found to be significantly increased in patients who did not require antihypertensive medication, possibly trying to compensate for the significant increase in LPO found in the determination at one year of follow-up. Some investigations mention the importance of mitochondrial deterioration and cell death due to apoptosis as a mechanism of nephrotoxicity manifested by an increase in LPO [31]. It was recently reported that circulating malondialdehyde concentration is independently associated with the long-term risk of cardiovascular mortality in RT recipients with relatively lower renal function [32].

In addition to traditional cardiovascular risk factors, the attention of RT recipients has focused on other complex pathophysiological processes related to posttransplant renal function, including the effect of OS. It is widely known that OS is increased in CKD and is further aggravated by renal replacement therapies. OS is associated with the appearance of atherosclerosis, left ventricular hypertrophy, and cardiorenal syndrome [26]. Various mechanisms have been proposed to explain the association between OS and atherosclerosis; among them are the following: (a) OS promotes enzymatic modification of circulating lipids and lipoproteins, (b) reactive oxygen species (ROS) are capable of directly conditioning dysfunction of endothelial [33], (c) immune system promotes a chronic proinflammatory state, and (d) OS promotes osteoblastic differentiation of vascular cells [34].

Indeed, the quality of life is low in patients with CKD; however, RT can improve the quality of life similar to healthy individuals [35]. The clinical and biochemical improvement observed in the RT recipients included in the present study is notable. However, some medications can delay kidney function in RT recipients, including adjusting immunosuppressive therapy, treating high blood pressure with ACEI drugs, ARA II type 1 drugs, calcium channel blocking drugs, and lipid control [36]. ACE inhibitors and type 1 ARBs may slow the progression of kidney disease by offering antiproteinuric effects [37, 38]. In 2013, the possible antioxidant protective effect related to the uptake of the Enalapril superoxide radical was reported by protecting the vascular endothelium against ROS in a dose-dependent manner in isolated abdominal aortas from rabbits and spontaneously hypertensive rats [39, 40].

Losartan is a potent, orally active, and selective nonpeptide blocker of the angiotensin II receptor type 1. Losartan reduces blood pressure, proteinuria, serum uric acid level, and posttransplant erythrocytosis. Losartan has positive effects on renal excretory function in adult CKD patients and RT recipients [41, 42]. Losartan increases proximal tubular caveolin 1. The intrarenal angiotensin II is probably involved in the downregulation of caveolin one during hypertension and kidney injury [43]. The HSP70i chaperone protein translocated to the plasma membrane, and its colocalization with caveolin one could be involved in the mechanism responsible for the cytoprotective effect of Losartan in the proximal tubules by decreasing OS through the downregulation of the NADPH oxidase Nox4 subunit [44]. However, these antihypertensives can have serious adverse events including hyperkalemia and metabolic acidosis [45]. In the present study, potassium levels did not change significantly in the final determination.

Various factors have been associated with increased cardiovascular risk in RT recipients during the postoperative period, such as the development of diabetes mellitus, arterial hypertension, dyslipidemia, and obesity [46]. Approximately 50% of patients gain weight after RT as a factor regardless of nutritional status before transplantation [47]. Increased body weight and its negative metabolic consequences may be associated with negative results after RT [48]. Per the preceding, in the present study, we found that patients who did not require antihypertensive medication and those who ingested Losartan significantly increased their weight at one year of follow-up, which could condition metabolic alterations with adverse effects evolution of RT.

The determination of sCr and urea and the estimation of GFR through different equations are considered biomarkers of kidney graft function used routinely in clinical transplantation. The sensitivity and specificity of these biomarkers are low, but they are inexpensive and readily accessible [49, 50]. In the present study, the levels of sCr and urea decreased significantly in the final determination as expected.

Another benefit of RT is the improvement in hemoglobin levels found in the three study groups in the determination at one year of follow-up under the previously reported. The authors found improvement in hemoglobin and hematocrit in patients with RT treated with TAC [51].

We consider that it is essential to monitor the expression of the oxidative damage marker to DNA, oxidants, and the activity of antioxidant enzymes as well as the function of the transplanted organ in addition to traditional kidney function tests to detect early alterations that can be corrected early to avoid the loss of the transplanted organ.

In conclusion, all the patients included in the study, regardless of the management with or without antihypertensive, presented overexpression of the marker of oxidative DNA damage, a significant increase in the activity of the SOD enzyme, and a significant decrease in the total antioxidant capacity as a systemic buffer of the redox state, which could suggest oxidative state imbalance. The increase in the activity of the SOD enzyme in all patients, including the increase in the GPx enzyme in patients who did not require antihypertensive medication, could suggest a compensatory effect in light of the significant increase in LPO in this group of patients. The present study results suggest that the determination of other markers in addition to those traditionally requested is required: sCr, urea, potassium, and glomerular filtration rate.

The limitations of the study are based on the small number of patients included and the short length of follow-up.

The study's strengths are based on the fact that it is a prospective cohort study before and after in patients with de novo RT with follow-up at one year. Soon, another study will be carried out with a longer follow-up time and a larger sample size.

## Figures and Tables

**Table 1:** Inherent data to renal transplantation.

	Not antihypertensive	Enalapril	Losartan	*p*
Age (years)	29 ± 3.83^a^	24.38 ± 1.61	28.46 ± 3.53	<0.01^∗^
Donor age (years)	37 ± 10.5	35.62 ± 7.32	39.54 ± 12.32	0.617
Transfusions	1.69 ± 0.48	1.62 ± 0.51	1.38 ± 0.51	0.273
Cold ischemia	56.46 ± 25.62	57.46 ± 17.08	70.01 ± 23.32	0.239
Warm ischemia	144.38 ± 101.41	122.62 ± 23.79	102.85 ± 19.21	0.237
Height (m)	1.68 ± 0.07	1.78 ± 0.08	1.72 ± 0.08^b^	<0.01^∗^

The results are expressed as mean ± standard deviation. ^a^vs. Enalapril. ^b^vs. Losartan. ^∗^ANOVA of a factor.

**Table 2:** Biochemical and anthropometric data.

	Not antihypertensive *n*-13			Enalapril *n*-13			Losartan *n*-13			
	Baseline	One year	WCX	Baseline	1 year	WCX	Baseline	One year	WCX	K-W
Weight (kg)	67.08 ± 11.51^b^	68.31 ± 10.94^b^	0.05^∗^	73.73 ± 12.93	74.46 ± 10.97	0.2	71.46 ± 13.11	74.08 ± 11.34	0.03^∗^	0.03^∗∗^
BMI (km/m^2^)	23.77 ± 3.56	23.97 ± 3.51	0.2	23.11 ± 3.37	23.32 ± 3.19	0.9	24.42 ± 3.63	25.27 ± 3.08	<0.01^∗^	0.05^∗∗^
SBP (mmHg)	119.23 ± 3.88^a,b^	122.54 ± 2.54^a,b^	<0.01^∗^	134.62 ± 7.54	127.8 ± 4	<0.01^∗^	140 ± 4.83	131.31 ± 7	<0.01^∗^	<0.01^∗∗^
DBP (mmHg)	78.62 ± 2.5^a,b^	76.62 ± 3^b^	0.1	87.31 ± 4.34	78.92 ± 1.5	<0.01^∗^	89.92 ± 5.48	80.77 ± 5.3	<0.01^∗^	<0.01^∗∗^
Glucose (mg/dL)	137.38 ± 80.42	90.46 ± 17.46	0.03^∗^	104.85 ± 31.48	87.08 ± 18.5	0.2	107.31 ± 35.03	103.23 ± 38.65	0.5	NS
Creatinine (mg/dL)	12.84 ± 3.77	1.18 ± 0.15	<0.01^∗^	12.16 ± 4.63	1.17 ± 0.21	<0.01^∗^	13.38 ± 1.79	2.6 ± 3.27	<0.01^∗^	NS
Urea (mg/dL)	122.92 ± 37.74^a^	31.39 ± 6.68	<0.01^∗^	155.59 ± 28.30	30.82 ± 5.05	<0.01^∗^	127.40 ± 32.11	70.04 ± 72.94	0.04^∗^	0.02^∗∗^
Potassium mmol/L	4.14 ± 0.63	4.10 ± 0.50	NS	4.33 ± 0.75	4.01 ± 0.50	NS	4.69 ± 0.68	4.47 ± 0.64	NS	NS
Leukocytes (%)	11.95 ± 4.87	8.76 ± 1.73	0.1	11.2 ± 8.78	9.03 ± 1.89	0.3	10.62 ± 2.89	8.86 ± 2.47	0.05^∗^	0.2
Lymphocytes (%)	11.08 ± 11.82^a^	22.16 ± 7.53	0.04^∗^	21.43 ± 10.73^b^	20.06 ± 8.4	0.5	8.87 ± 4.45	15.93 ± 7.18	<0.01^∗^	<0.01^∗∗^
Hemoglobin (mg/dL)	11.14 ± 1.45	12.36 ± 0.69	<0.01^∗^	10.23 ± 1.88	12.58 ± 34.38	<0.01^∗^	10.58 ± 1.57	12.17 ± 1.61	0.02^∗^	NS
Hematocrit (%)	35.28 ± 4.64	38.52 ± 3.01	0.6	34.75 ± 9.98	37.94 ± 3.68	0.1	31.88 ± 3.2	37.05 ± 5.59	<0.01^∗^	NS
Platelets (*μ*L)	230.62 ± 48.25^a,b^	283.46 ± 51.5	<0.01^∗^	282.46 ± 25.46	322.69 ± 59.3	0.1	295.46 ± 41.56	331.77 ± 71.13	0.02^∗^	<0.01^∗∗^
TAC levels	10.24 ± 7.33	14.78 ± 8.06	NS	8.88 ± 5.03	11.32 ± 1.47	NS	10.29 ± 4.3	11.92 ± 4.4	NS	NS

The results are expressed as mean ± standard deviation. ^a^vs. Enalapril. ^b^vs. Losartan. ^∗^Wilcoxon rank test. ^∗∗^Kruskal-Wallis test. ^a^,^b^The different letter denotes statistically significant difference using the Dunn-Bonferroni paired test. BMI: body mass index; SBP: systolic blood pressure; DBP: diastolic blood pressure; NS: nonsignificant.

**Table 3:** Oxidative stress markers.

	Not antihypertensive *n*-13			Enalapril *n*-13			Losartan *n*-13	
	Baseline	One year	WCX	Baseline	One year	WCX	Baseline	One year
*Oxidants*								
LPO (mM)	5.6 ± 2.61	8.65 ± 1.09	<0.01^∗^	5.08 ± 3.11	9.5 ± 2.68	0.1	8.73 ± 8.6	7.45 ± 3.81
NO (*μ*g/mL)	374.56 ± 42.97	350.8 ± 111.32	0.8	382.8 ± 42.06	343.51 ± 138.46	0.3	352.2 ± 47.46	401.51 ± 72.06
*Oxidative damage to DNA*								
8-OHdG (ng/mL)	68.88 ± 11^a^	74.47 ± 0.39	<0.01^∗^	52.94 ± 21.45	74.29 ± 0.63	0.02^∗^	62.71 ± 19.6	73.78 ± 1.88
*Antioxidants*								
SOD (U/L)	0.3 ± 0.2	1.17 ± 0.63	<0.01^∗^	0.39 ± 0.17	1.31 ± 0.67	<0.01^∗^	0.25 ± 0.21	1.19 ± 0.46
GPx (nmol/min/mL)	343.76 ± 214.82	518.15 ± 163	0.03^∗^	288.70 ± 212.12	493.1 ± 264.06	0.9	448.61 ± 146.96	442.3 ± 242.67
TAC (*μ*M)	59.78 ± 1.89	17.52 ± 3.4	<0.01^∗^	65.45 ± 18.99	14.2 ± 3.2^b^	<0.01^∗^	61.10 ± 24.07	20.7 ± 5.3

The results are expressed as mean ± standard deviation. ^a^vs. Enalapril. ^b^vs. Losartan. ^∗^Wilcoxon rank test. ^∗∗^Kruskal-Wallis test. ^a,b^The different letter denotes statistical significant difference using the Dunn-Bonferroni paired test. LPO: lipoperoxides; NO: nitric oxide; 8-OHdG: 8-hydroxy2′-deoxyguanosine; SOD: superoxide dismutase; GPx: glutathione peroxidase. TAC: total antioxidant capacity.

## Data Availability

Clinical data is restricted to protect the confidentiality of the participants and against misuse of the information. The data may be shared by the Mexican Social Security Institute (IMSS) through the approval of the Institution's Ethics Committee. Data used to support the findings of this study will be made available by the corresponding author provided the criteria for access to confidential data.
